# Interpersonal and systemic factors in initiating, developing and maintaining collaborations between European neurosurgical departments and institutions in low-resources settings: A qualitative study

**DOI:** 10.1016/j.bas.2025.104303

**Published:** 2025-06-19

**Authors:** Nicolò Marchesini, Vicki M. Butenschoen, Andreas K. Demetriades, Said Idrissa Ahmada, Fazlul Hoque, Thomas Kapapa, Patrick D. Kamalo, Pablo González-López, Rupavathana Mahesperan, Ondra Petr, Wilco Peul, Nicephorus Boniface Rutabasibwa, Ellianne J. dos Santos Rubio, Abenezer Tirsit Aklilu, Jake Timothy, Enoch O. Uche, Magnus Tisell

**Affiliations:** a Global and Humanitarian Neurosurgery Committee, European Association of Neurosurgical Societies (EANS), Brussels, Belgium; b Department of Neurosurgery, Azienda Ospedaliera Universitaria Integrata di Verona, Verona, Italy; c Leiden University Medical Center (LUMC), Leiden, the Netherlands; d Department of Neurosurgery, Technical University Munich, School of Medicine, Munich, Germany; e Department of Neurosurgery, Royal Infirmary Edinburgh, NHS Lothian, Edinburgh, United Kingdom; f Department of Neurosurgery (NED Institute), Mnazi MMoja Hospital, Zanzibar, Tanzania; g Square Hospital, Dacca, Bangladesh; h Department of Neurosurgery, Ulm University Hospital, Ulm, Germany; i Blantyre Institute of Neurological Sciences, Department of Neurosurgery, Queen Elizabeth Central Hospital, Ministry of Health, Blantyre, Malawi; j Department of Neurosurgery, Hospital General Universitario Alicante, Alicante, Spain; k Department of Optics, Pharmacology and Anatomy, University of Alicante, Alicante, Spain; l Department of Neurosurgery, Haukeland University, Bergen, Norway; m Department of Neurosurgery, Medical University Innsbruck, Innsbruck, Austria; n Department of Neurosurgery, University Neurosurgical Center Holland, UMC | HMC | HAGA, Leiden, The Hague, the Netherlands; o Department of Neurosurgery, Muhimbili Orthopaedic Institute (MOI), Dar es Salaam, Tanzania; p Department of Neurosurgery, Curaçao Medical Center, Willemstad, Curaçao; q Division of Neurosurgery, Department of Surgery, Addis Ababa University, Addis Ababa, Ethiopia; r Department of Neurosurgery, Leeds General Infirmary, Leeds, United Kingdom; s Division of Neurosurgery, College of Medicine, University of Nigeria Nsukka, Ituku, Enugu, Nigeria; t Department of Neurosurgery, University of Gothenburg, Gothenburg, Sweden

**Keywords:** Global neurosurgery, Global surgery, Global health, Low- and middle-income countries, Collaboration, Qualitative study

## Abstract

**Introduction:**

Neurosurgical care in low- and middle-income countries faces persistent challenges, including insufficient infrastructure, lack of trained surgeons, and limited access to sustainable training programs. Collaborative initiatives with high-income countries aim to address these gaps. However, in-depth studies of European-led partnerships and the interpersonal and systemic factors underpinning their success remain limited.

**Research question:**

What are the most salient interpersonal and systemic factors relevant to the a) initiation, b) development, and c) maintenance of effective and sustainable collaborations between European neurosurgical departments and institutions in resource-limited settings?

**Material and methods:**

We conducted a prospective qualitative study using semi-structured interviews with fourteen matched neurosurgeons—seven from European centers and seven from LMIC institutions—engaged in such collaborations. Data were collected virtually between May and August 2024. Transcripts were analyzed thematically to identify major themes, which were coded and categorized.

**Results:**

Twelve themes emerged, grouped into three stages: a) initiation: trust and personal connections, systematic planning and foundations, local context and needs, institutional and government support; b) development: capacity building and skills development, academic and research growth, challenges to development and adaptation; c) maintenance: sustainability and independence, resources and logistical support, communication and continuous commitment, outcome measures and accountability, and challenges to maintenance and advices for continuity.

**Discussion and conclusions:**

Findings highlight recurring interpersonal and systemic dynamics central to successful long-term partnerships. This study provides context-specific, real-world insights into their practical execution. Future efforts should focus on developing targeted recommendations to strengthen global neurosurgical collaborations and address inequities in access to care.

## Highlights

• Interviews explore neurosurgical collaborations lasting over five years.
• Results reveal twelve key themes across initiation, development, and maintenance.
• Study emphasizes bidirectional partnerships and cultural humility.
• Trust, adaptability, and sustainability are essential for long-term impact.

## List of abbreviations

HICs: High-income countries
LMICs: Low- and middle-income countries
L-MICs: lower-middle income countries
LICs: low-income countries
NGOs: non-governmental organizations

## 1 Background

In limited resource settings, neurosurgical care is highly inequitable, and global neurosurgery is receiving increasing attention. Many low- and middle-income countries (LMICs) lack infrastructure, equipment and utilities and do not have an adequate number of trained surgeons to fulfill their neurosurgical needs, leading to a high burden of neurosurgical diseases (Garcia et al., 2024; Haizel-Cobbina et al., 2024; Weiss et al., 2019; GBD, 2016 Traumatic Brain Injury and Spinal Cord Injury Collaborators, 2019). Furthermore, LMICs face significant challenges due to the loss of trained professionals to migration ('brain drain') and financial constraints. Finally, the high incidence of trauma, infectious diseases, and cultural beliefs, with a lack of governmental healthcare awareness and rehabilitation options, contribute to the inefficiency of neurosurgical care.

Many efforts have been made to address these challenges, including bilateral high-income countries (HICs)-LMICs programs and cooperative initiatives. In this process, establishing self-sustainable collaborations is an essential element to enhance the autonomy of the LMICs partners and reduce their dependence on HICs (Garcia et al., 2024; Fuller et al., 2021; Jelmoni et al., 2024; Marchesini et al., 2022, 2024). In other words, collaborations are deemed successful if self-sustainable programs can be established.

While the overall interest in these partnerships is increasing, barriers and pitfalls still exist (Marchesini et al., 2022). First, published research focuses either on systematic reviews depicting the currently available programs or describes the outcome of specific programs in designated LMICs (Weiss et al., 2019; Wicaksono et al., 2020; Budohoski et al., 2018; Okon et al., 2024). Secondly, specific information about collaborations involving the European global neurosurgical landscape are lacking (Fuller et al., 2021). Finally, crucial parameters to ensure a project's success and practical recommendations learned through experience are missing. This type of information cannot be fully captured through quantitative research alone, necessitating a more comprehensive and reflexive methodological approach. For this reason, we adopted a qualitative approach, which is well-suited to exploring the nuanced interpersonal and systemic dynamics involved. This methodology allows for an in-depth understanding of participants’ lived experiences and facilitates the emergence of themes that may not be evident through quantitative means.

We hereby present the results of our study highlighting the experience of key stakeholders from European neurosurgical Institutions and their matched partners in contexts with limited resources, to capture diverse and meaningful lessons learned from engaging in successful international partnerships. Our primary focus is on the initiation, development, and maintenance of such collaborations, with an emphasis on exploring the challenges and achievements critical to the project's success.

## 2 Research question

What are the most salient interpersonal and systemic factors relevant for the a) initiation, b) development, and c) maintenance of effective and sustainable collaborations between European neurosurgical departments/organizations and Institutions/organizations in context with limited resources?

## 3 Methods

### 3.1 Research design and theoretical framework

This study employed a prospective qualitative research design using semi-structured interviews to answer the research questions. The research was grounded in a descriptive qualitative approach, which guided the data collection and analysis processes.

### 3.2 Research team and reflexivity

The core research team (NM, VB, EU, MT) consisted of researchers with experience in global neurosurgery and international collaborations. The primary researchers (NM and VB) conducted all interviews and were not personally involved in any of the collaborations. To ensure reflexivity, the team engaged in team discussions to identify and mitigate potential biases stemming from their professional role or personal experiences.

### 3.3 Participant selection

Participants were selected using purposive sampling based on their direct involvement in international neurosurgical collaborations lasting five years or more. Eligibility criteria included: (1) holding a clinical role as a neurosurgeon; (2) having a leadership or foundational role in the partnership; and (3) willingness to participate in a semi-structured interview. Participants were initially approached by email and all accepted to participate. Individuals from both European institutions and their LMIC partners were included to ensure balanced representation and promote bilateral insights. Potential participants were identified through professional networks and institutional referrals. We acknowledge that focusing solely on neurosurgeons may omit other critical perspectives; this decision was made to ensure a focused and comparable set of experiences but future research should broaden the participant base. All participants provided informed consent before participating in the study. Data saturation was expected to be reached by interviewing 12–18 candidates.

### 3.4 Data collection

Data was collected between May and August 2024. Semi-structured virtual interviews were conducted using an interview guide developed by NM, VB, MT and EU. The guide included closed and open-ended questions (Supplement I). An academic with prior experience in qualitative research on global surgery supervised the process. NM and VB did not have any previous personal relationships with the interviewees. An interview-guide was sent to participants immediately before the interview, to ensure adequate comprehension. After two pilot interviews attended by both NM and VB, the remaining interviews were conducted individually online via video conferencing (Zoom Video Communications, San José, California). Interviews lasted between 26 and 93 min (mean 58, median 57) excluding initial introductions and post-interview conversations. All interviews were audio-recorded with participants’ consent and transcribed verbatim. Field notes were taken to document non-verbal cues and contextual information. All interviewees were given the opportunity to opt out of the interview or to refuse to answer questions if deemed inappropriate. The accuracy of the transcripts was cross-checked by both researchers, who reviewed each other's interviews by listening to the recordings.

### 3.5 Ethical considerations

The study was conducted in accordance with the ethical principles of the Declaration of Helsinki. Ethical approval was obtained (GHC250324). Informed consent was obtained from all participants prior to data collection, and participants were assured of confidentiality and their right to withdraw from the study at any time without consequence. All interviews were conducted with participants' explicit consent, and their anonymity was maintained throughout the research process. Data confidentiality was maintained by de-identifying transcripts and storing them on a safe server.

### 3.6 Data analysis

Thematic analysis was conducted by NM and VB following the framework proposed by Braun and Clarke (2006). Transcripts were initially read multiple times to achieve immersion by both researchers. A coding framework was developed iteratively by open coding. To ensure rigor while achieving richer interpretation, the analysis involved double-coding by NM and VB with several iterations of coding and adjustments. Themes, codes and relevant quotes were shared with MT and EU for refinement. Discrepancies were resolved through discussions. Codes were then grouped into themes based on patterns and relationships within the data. The open-source qualitative data analysis tool *Taguette* (https://www.taguette.org/) was used to organize and manage the data. The final codes and themes were shared with the interviewees, who were invited to provide additional insights.

### 3.7 Reporting

The findings are reported following the COREQ checklist to ensure transparency and comprehensiveness when applicable (Supplement II) (Tong et al., 2007).

## 4 Results

In total, 14 surgeons with diverse experiences in collaborations were interviewed. Seven participants were affiliated with European institutions, while the other seven were affiliated with institutions in LMICs (World Bank). An exception was Curaçao: although officially classified as a high-income country (HIC), its poverty rate exceeds 30 %, and the operational context of the solo neurosurgeon on the Caribbean island was deemed suitable for the study's purposes (Vordev, 2024). Complete participant and collaboration characteristics are presented in Table 1. Relevant quotes are reported as stated by a European (E) or low-resource setting interviewee (L) in Table 2, Table 3, Table 4.

**Table 1:** Participant details as declared by the interviewees. Years of experience refers to the years of clinical practice after the completion of specialization, while years in the collaboration refers to the number of years the participant was directly involved in the partnership. The order in which the data are presented corresponds to the paired institutions.

Participant	Institution Name	Institution type	Country	Income region	Years of experience	Years in the collaboration
1	Sahlgrenska University Hospital	Academic Medical Center/University Hospital	Sweden	HIC	27	7
2	University of Nigeria Teaching Hospital Enugu	Academic Medical Center/University Hospital	Nigeria	L-MIC	15	10
3	Ulm University Hospital	Academic Medical Center/University Hospital	Germany	HIC	16	8
4	Queen Elizabeth Central Hospital	Teaching Hospital (non-university)	Malawi	LIC	14	12
5	University of Innsbruck	Academic Medical Center/University Hospital	Austria	HIC	13	10
6	Muhimbili Orthopaedic Institute (MOI)	Academic Medical Center/University Hospital	Tanzania	L-MIC	13	12
7	Leiden University Medical Center & The Hague Medical Center	Academic Medical Center/University Hospital	The Netherlands	HIC	25	10
8	Curaçao Medical Center	Government/Public Hospital	Curaçao	HIC	9	8
9	Hospital General Universitario de Alicante	Academic Medical Center/University Hospital	Spain	HIC	12	9
10	NED Institute Mnazi Mmoja Hospital	Government/Public Hospital	Zanzibar	L-MIC	8	5
11	Leeds Teaching Hospital NHS Trust	Teaching Hospital (non-university)	UK	HIC	22	22
12	Square Hospital, Dacca	Private Hospital	Bangladesh	L-MIC	35	22
13	Department of Neurosurgery, Haukeland University	Academic Medical Center/University Hospital	Norway	HIC	20	17
14	Division of Neurosurgery, Addis Ababa University	Academic Medical Center/University Hospital	Ethiopia	LIC	10	15

**Table 2:** Relevant quotes of the respondents for the initiation phase, themes 1 to 4. Quotes are presented as stated by a european or low-resource setting interviewee.

Initiation		
	European respondent	Low-resources respondent
Theme 1: trust and personal connection	● Build a strong relationship with your partner … is the most important thing, without this, it's not possible ● It was very important for me, that they also become friends	● What can give you a strong foundation? Trust. And building trust takes time ● And such opportunities should be informal first, and then you spend time developing trust rather than rushing into collaborations ● Go there and experience it yourself, really dive in ● Bi-directionality means that there are certain rules … it's not a question of a unilateral relationship where decisions are taken from one center and the other just has to comply
Theme 2: systematic planning and foundations		● What may work in [one country] may not work in [another] ● It is also important to have timelines.It doesn't have to be closed timelines. Things can be reevaluated and … modified ● You start with what is possible … you scale up to what is probable … as collaborations become more successful.complex tasks become easier ● If we plan together and everyone's expectations are known … It will make things easier ● We also looked at their templates … and we modified it to suit the neurosurgical compartment
Theme 3: local context and needs	● Talk with the other side about the needs … about the possible goals ● It should be a person in an executive position … who has the power to make decisions ● The first thing you have to do is try to understand a different culture … understanding, listening	● We also looked at the infrastructure that was required to make the whole collaboration … identified the critical stakeholders
Theme 4: institutional and government support	● Involving the Federal Ministry of Health at the beginning … would have made our collaboration a national collaboration.and it would have made certain things.easier to handle ● You need to establish contact with high-level officials as early as possible, ideally with the Health Minister	● NGOs, universities, and private institutes are also part of giving better attention to the field. It has an enormous impact … in advocacy, prevention, and public awareness

**Table 3:** Relevant quotes of the respondents for the development phase, themes 5 to 7. Quotes are presented as stated by a european or low-resource setting interviewee.

Development		
	European respondent	Low-resources respondent
Theme 5: capacity building and skills development	● You need to look at the other parts, like the nursing team, rehabilitation, and so forth … you can be the best surgeon in the world, and the patients will die as flies if you don't have good postoperative care ● You should build the capacity and knowledge sharing through training program workshops, even fellowships or online courses ● If you have the possibility to motivate them, you should train the trainers	● If I can share the knowledge with them, they will learn … it will be disseminated to the different parts of the country, and the country's people will benefit ● When they come back you can see that they interplayed something. So behaviors change, practices change, attitudes change
Theme 6: academic and research growth	● You should try to establish the research not only for your European institution but also for them, because then they can also provide and show how active they are, and they can then apply for funding	● You can't just have a collaboration that doesn't have a foundational scientific concept ● That link with academics is important, as well as for research and capacity building ● In order to publish in a high-impact journal … you need someone you are collaborating with … it's very difficult to get the editors to take your publication here … But if you have someone involved with you who is collaborating with you, then it is much more easier
Theme 7: challenges to development and adaptation	● In one of our missions, suddenly they decided to close the airport ● Patients who have no money have to buy expensive implants … these are the challenges of creating a sustainable situation ● The lesson I learned is maybe that I have to think more about my personal structure. Maybe I'm too structured … I have to think about loosening the knots and look at what's happening ● my barrier was my flexibility, my rigidity on the way in which I was thinking	● The electricity here is not stable. I did not know that when I came here. It broke down a lot of the things that I used ● Sometimes we are operating in Africa, and they take the light. They do that all the time ● We have an enormous problem. Our number is increasing. However, the infrastructure is not developing much … and the diversity and complexity are also increasing ● They have a very high health illiteracy here … they don't know what medications they take

**Table 4:** Relevant quotes of the respondents for the maintenance phase, themes 8 to 12. Quotes are presented as stated by a european or low-resource setting interviewee.

Maintenance		
	European respondent	Low-resources respondent
Theme 8: sustainability and independence	● We do think that the collaboration should come to an end, we should be able to maintain most of the services that we have established through the collaboration ● We've been given some technology that we need … licenses for certain things … if assuming the information cut, what will happen to that?	● We do think that the collaboration should come to an end, we should be able to maintain most of the services that we have established through the collaboration ● You should have a timeline that a unit that is a recipient today … can become a donor tomorrow ● We've been given some technology that we need … licenses for certain things … if assuming the information cut, what will happen to that?
Theme 9: resources and logistical support	● Operate in their own environment with their own equipment, and make the best you can do with that ● We try to make it as targeted as possible, if you go ask our collaborators, it's a long list … we always try to rank … it's impossible to fulfill all the needs ● The second time I went over, the drills … were on the shelf gathering dust ● If something is donated, then we come down and show it a little bit how to maintain it and how to use it ● The infrastructure always lags behind human resource development. If there is anything we can do from the initial planning to help us accelerate the infrastructure development, that would have been better	● The collaboration helps to open the eyes of the department in the government that this one can be done, and we need this one
Theme 10: communication and continuous efforts	● We need to remain friends … so that we can continue working together … we need to manage [issues] so that both parties are happy … and would want to stay in ● If you stop communicating … one will never be able to transfer the vision ● It's a child. It's a growing child, and no child grows up properly if you leave it like that. It causes problems. But you are there to solve the problems. It's a very productive child. A child with two parents, and sometimes one parent has to do more, and the other time it's the way around ● It's not a program for one year … it will be ten years … you should start, and you should finish it	● Communication has to be open ● You need an environment where people can communicate freely, and if they present their side, we should be prepared to accommodate ● It should not just be one side accommodating … both sides should accommodate ● Every extra effort you put in to ensure that you move the needle of care will get a very great reward if you do that. So don't get fatigued, or just say “you know, this is not possible”. Just keep pushing … it translates to important outcomes for the patients
Theme 11: outcome measures and accountability	● We developed or have been developing standardized protocols based on our own results ● Complications down there are much worse … because they're sometimes fatal. So the more you can do to lower the complications the better ● You have to be aware, as a European guy, that the local guys do this pre-selection without you. You have to live with it, you know. The ethical part is very difficult	● Our outcome has to be measured … to maintain the quality of the service and the training ● You also have to stop sometimes to measure and see what we achieved so far.and to see what you need to improve … to see if you are going in the right direction ● We need to meticulously see their exposure, and if they're getting an adequate number of cases and if they're exposed to a variety of cases ● “… we have exams, and during the exams, we invite external examiners who will help us assess the strength of the training programs
Theme 12: challenges to maintenance and advice for continuity	● The fatigue of collaboration … becomes monotonous. A way we can … address this is to expand the field … as more people get involved, then we can really be able to … amplify the impact ● The only problem is the bureaucracy … It never used to be so bureaucratic in the past. But now they have to go through a lot ● All your expenses are coming from my pocket, my trip and my meals there ● If they have a condition that everybody has gone on their free time paying for themselves, I think it's harder to get it sustainable ● Now, for example, that I have a kid … I'm taking two weeks off from my family	● Human resources rapidly increase, but the available infrastructure does not … So that is frustrating … some of them leave for neighboring countries ● The challenge was the funding. Because when you organize these lectures, you need to have transportation … you need to convince people from abroad … and we needed to have some … budget

After qualitative data analysis, twelve themes were identified: (1) Trust and personal connections, (2) Systematic planning and foundations, (3) Local context and needs, (4) Institutional and government support, (5) Capacity building and skills development, (6) Academic and research growth, (7) Challenges to development and adaptation, (8) Sustainability and independence, (9) Resources and logistical support, (10) Communication and continuous commitment, (11) Outcome measures and accountability, and (12) Challenges to maintenance and advice for continuity.

**Fig. 1:**
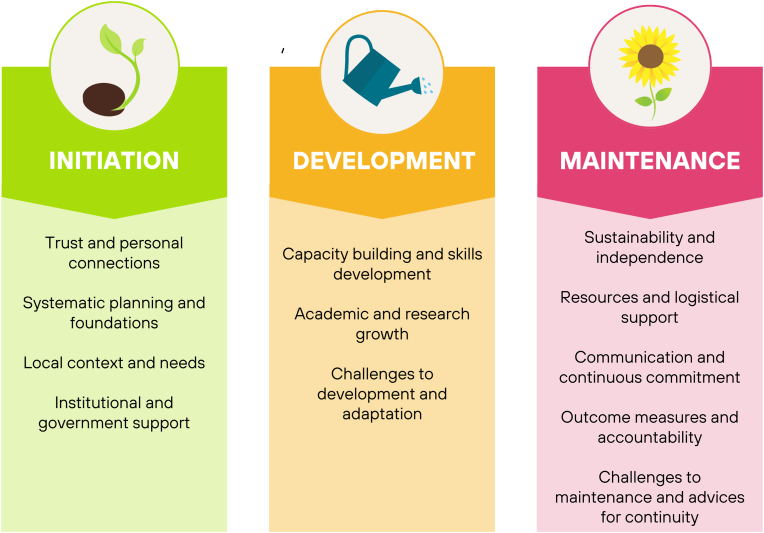
Themes identified through qualitative data analysis, categorized into three overarching stages of collaboration: initiation, development, and maintenance. Each theme reflects key factors relevant to effective and sustainable partnerships between European neurosurgical departments and institutions in low-resource settings.

Aligned with the research questions, the themes were categorized into three overarching categories: initiation (themes 1 to 4), development (themes 5 to 7), and maintenance (themes 8 to 12) (Fig. 1).

## 5 Initiation

### 5.1 Theme 1: trust and personal connection

Mutual trust and genuine personal connections represent essential prerequisites for successful collaborations. However, trust is a gradual process that should not be rushed, requires time, mutual respect, and repeated positive interactions. Conferences, workshops and other meetings are examples of opportunities to initially meet each other and build trust. Such collaborations should not rely on merely professional transactions, but rather on meaningful interpersonal relationships. Immersive experiences, such as spending time on-site and connecting with local partners on a deeper level, emerged as pivotal to building empathy and understanding, which ultimately supports long-lasting commitment. Finally, sharing responsibilities and decisions since inception is essential to fostering an environment of equality between partners.

### 5.2 Theme 2: systematic planning and foundations

Structured and strategic planning are pivotal elements and the formulation of a *memorandum of understanding*, a collaboratively designed framework, may help to map out a clear strategy based on specific contextual needs. Systematic planning also includes setting achievable timelines and flexible goals that allow for gradual expansion of the collaboration. Shared planning allows to clearly define responsibilities, fostering the development of a cooperative environment. Adopting templates and strategies borrowed from other successful efforts, customized for neurosurgical needs, may be an effective approach. In general, systematic and cooperative planning offers a distinct pathway for building resilience, allowing partnerships to become more flexible in response to challenges that may arise over time.

### 5.3 Theme 3: local context and needs

A thorough exploration and understanding of the local context and specific needs is paramount and infrastructures, resources, and key stakeholders should be clearly identified at the outset. Recognizing local decision-making dynamics is important to respect the autonomy of the partner institution. Additionally, early involvement of individuals in executive roles may help to ensure that collaborations are supported at an institutional level and not only by operative individuals. The appreciation of local context and needs not only facilitates the practical aspects of collaboration but also builds rapport and respect that can be the ground for long term partnerships.

### 5.4 Theme 4: institutional and government support

Institutional and governmental support may play a significant role in initiating such types of partnerships. In detail, engaging government bodies, such as the Ministry of Health, from the outset can shift the collaboration from hospital-level to broader national alliances. While this process may strengthen institutional commitment, it can also open doors to resources and other logistical facilitations. The engagement of high-level government figures can be important to secure strategic oversight in fields such as policy support, reinforcing the collaboration's sustainability since the initiation. Other players can be supportive at this stage of the collaboration, such as non-governmental organizations (NGOs), universities, and private entities. Their roles may include raising public awareness, conducting advocacy and providing supplementary resources.

## 6 Development

### 6.1 Theme 5: capacity building and skills development

Capacity building and skills development are essential for sustainable partnerships, requiring involvement from neurosurgeons and other healthcare professionals across all levels. Sustainability demands that educational efforts be structured and ongoing, with various options available. Empowering local professionals to continue skill development independently is crucial, achieved through “training the trainers.” Although local manpower is available, specialized skills are often lacking, making partnerships vital for enhancing competencies and expertise. These benefits can reach beyond the immediate region through a cascade effect. Training can also involve bidirectional exchanges, fostering resilience in LMIC health systems by influencing practices and attitudes.

### 6.2 Theme 6: academic and research growth

Academic and research growth is crucial at this stage of collaboration, which should be itself rooted in a scientific framework. *.* Building an academic foundation engages both partners in medical advancements and research trends, fostering mutual clinical and research progress. A collaborative research environment should allow both institutions to actively contribute to studies and publications and support not only European institutions' research agendas but also empower LMIC institutions to increase visibility and access to funding. Such partnerships may also enhance LMIC institutions' presence in high-impact journals.

### 6.3 Theme 7: challenges to development and adaptation

Collaboration progress can be hindered by various systemic and environmental factors and anticipating, recognizing, and addressing these challenges can strengthen partnerships. A recurring theme is the need to adapt to infrastructure constraints. Unpredictable local logistics can further complicate clinical activities. Financial barriers also limit collaborative progress, especially for patients who must often purchase their own costly, substandard implants, posing ethical and logistical challenges. Infrastructure challenges also arise with growth*.* Cultural differences further complicate interactions. Overcoming these challenges demands personal adaptability, flexibility, and mutual understanding.

## 7 Maintenance

### 7.1 Theme 8: sustainability and independence

Achieving local team autonomy is essential for sustainable programs, though balancing support and independence is challenging. Local institutions should gradually uphold services initiated through partnerships, which requires a joint commitment to building local capacity and planning for self-sufficiency. This goal requires reducing strict oversight, allowing local teams to operate independently. Sustaining independence is complex and should not rely on a few individuals but instead require broad local support, to reduce the risk of failure upon their exit. Beyond personnel, technology that initially aids development can later hinder independence if not planned strategically. Therefore, transitioning towards self-reliance requires a structured but flexible approach, ensuring both technological and operational independence for sustained impact.

### 7.2 Theme 9: resources and logistical support

Effective resource allocation and maintenance are vital for successful partnerships. Identifying and providing critical equipment with a significant impact on neurosurgical capacity often requires substantial financial investment and infrastructure support. A practical approach is to begin with existing facilities. Donated equipment should align with local capabilities and real needs, given that demand often exceeds feasible supply. Maintenance remains a significant challenge due to limited infrastructure and training to handle specialized equipment. Training on the use and maintenance of donated equipment may address this issue. This approach strengthens technical capacity and sustains the long-term functionality of equipment. Addressing logistical chains and infrastructure at the outset can promote sustainability. Finally, collaborations can raise government awareness of resource needs.

### 7.3 Theme 10: communication and continuous efforts

Robust communication and unwavering commitment are fundamental for effective partnerships. Open dialogue ensures alignment and allows proactive problem-solving. Such openness prevents crises from threatening the partnership and can even strengthen bonds. Consistent communication is also vital for maintaining a shared vision*.* Mutual respect and adaptability are key-factors. Despite challenges like fatigue or resignation, persistent effort yields significant rewards*.* Finally, commitment to partnership should be maintained in a long-term perspective.

### 7.4 Theme 11: outcome measures and accountability

The implementation of consistent outcome measures and accountability frameworks is essential. They ensure the quality while maintaining rigorous standards. Regular self-assessment fosters adaptability to evolving needs. Practical examples include creating standardized protocols or standard examination of clinical exposure. Standards ensure trainees receive comprehensive education. These measures must address local challenges, especially in resource-limited settings. Furthermore, European partners must respect local practices, even when ethical considerations may differ. Integrating these accountability measures within local contexts fosters the maintenance of sustainable and respectful partnerships.

### 7.5 Theme 12: challenges to maintenance and advices for continuity

Several challenges undermine the maintenance of such partnerships. Limited infrastructure relative to growing human resources is a major issue. This mismatch can create frustration, prompting personnel to seek opportunities elsewhere and threatening program continuity. Monotony and lack of new stimuli can also lead to burnout, reducing enthusiasm. Expanding activities and involving more team members can mitigate this issue. Bureaucracy and legal challenges complicate efforts for international surgeons. Funding is another reported challenge across all levels*.* Some expressed that they bear personal expenses, including travel and accommodation, making sustained involvement more difficult*.* However, this may affect sustainability*.* Family obligations and personal responsibilities also impact commitment. Addressing these issues early is crucial for ensuring long-term sustainability and impact.

## 8 Discussion

This study offers novel insights by integrating the bilateral reflections of neurosurgeons across long-standing collaborations, emphasizing interpersonal and systemic factors in equal measure. Unlike prior literature that often highlights one-sided or time-limited efforts, our study showcases the evolution of trust, adaptability, and sustainability over time. Importantly, it positions these insights within a European context, which has been underexplored compared to North American counterparts. Moreover, our findings offer a nuanced understanding of collaboration as a dynamic and evolving process. Rather than listing generic success factors, we present context-specific examples that illustrate how collaboration is co-constructed, iteratively adapted, and deeply reliant on human connection. While at first glance some of the themes identified in this study may appear intuitive or self-evident, our findings highlight how these principles take on complex, context-dependent forms in the reality of long-term global surgical partnerships. The nuanced narratives provided by experienced neurosurgeons show that success is not simply a matter of applying standard collaboration models, but rather involves continuous negotiation, cultural humility, and adaptive learning—insights that are often underestimated or insufficiently described in prior literature. To summarise, this study explored key interpersonal and systemic factors influencing the initiation, development, and maintenance of collaborations between European neurosurgical Departments and Institutions in contexts with limited resources. Through interviews with surgeons with diverse experience in these collaborations, we identified twelve overarching themes that collectively shed light on the essential components of such activities. Additionally, the results may offer an evidence-based foundation to inform the development of recommendations for effective, sustainable international collaborations between European Departments and Institutions in LMICs, beyond the field of neurosurgery.

The results of our study align with the literature emphasizing the necessity of ensuring equity and ethics in partnerships between HICs and LMICs. While ensuring equitable access to safe and effective surgical care remains a critical aspect in the Global Health agenda, these partnerships are increasingly recognized as essential for addressing such disparities (Bentounsi and Nazir, 2020). Various models of collaboration including surgical missions, long-term partnerships, and fellowship training, have been documented (Almeida et al., 2018). Our study highlights the importance of adapting these models to local contexts and needs, emphasizing the personal experiences of clinicians actively engaged in these initiatives.

Our results confirm that the initiation of collaborations must be based on trust and personal connections. The foundational role of trust in global health partnerships has been highlighted by other authors, who argued that long-term collaborations demand "relational ethics" where moral commitments precede legal or contractual ones (Kerasidou et al., 2021). In our study, participants emphasized that trust was not just a facilitator but a prerequisite, evolving through informal interactions and sustained exposure. This aligns with recent recommendations that prioritize mutual accountability and interpersonal transparency as necessary conditions for ethical North-South partnerships.

Moreover, systematic planning and sensitivity to local contexts were highlighted, with participants emphasizing the relevance of aligning goals and objectives with local needs through formalized agreements (Greive-Price et al., 2020). These findings confirm the critical role of cultural sensitivity for successful partnerships.

In the development phase of collaborations, increasing local capacity and technical and academic skills were reported as pivotal elements. This reflects the global health imperative to strengthen local healthcare systems (Fuller et al., 2021). Along with clinical development, our results align with prior studies showing that research collaborations can help enhance clinical capacities but also may result in high-quality studies led by academics in LMICs (Wu et al., 2017). This emphasis is consistent with Rubiano et al., who described how North-South collaborations in neurotrauma led to locally-run fellowship programs supported by international academic mentorship (Rubiano et al., 2021). Similarly, Almeida et al. underscored the role of bidirectional learning and training in their account of international neurosurgical education at a U.S. university, showing that both LMIC and HIC professionals benefit from shared exposure to diverse clinical environments (Almeida et al., 2018). These findings reinforce the need for sustained educational infrastructure and underscore that capacity building must extend beyond short-term missions. Our participants confirmed the need for long-term training initiatives and academic programs that prioritize local empowerment over short-term interventions. However, consistently with previous studies, several challenges that may hinder such initiatives were described (Marchesini et al., 2024; Almeida et al., 2018; Establishing Translational and Clinical Cancer).

Aiming to self-sustainability and independence was highlighted as the key goal to ensure long-term collaborations. The progressive reduction of dependence on HIC partners was one of the most important elements that our participants reported. Successful models where subspecialized training programs are implemented in LMICs while maintaining international academic support exist in the literature (Rubiano et al., 2021). However, program sustainability requires deliberate transition planning. In their systematic review, Greive-Price et al. found that the absence of planned exits and handovers often led to stalled partnerships (Greive-Price et al., 2020). By contrast, our participants’ emphasis on autonomy and their view of LMIC institutions as potential future donors demonstrates a paradigm shift toward balanced reciprocity and dynamic leadership roles over time.

Power asymmetries remain a persistent concern in global health collaborations. Despite discourses of equality, many partnerships are governed by unspoken hierarchies and implicit donor dominance. Our participants repeatedly emphasized the importance of local leadership in agenda setting, echoing recent calls for a decolonized global health approach that prioritizes local agency and contextual authority (Abimbola, 2019). Structuring collaborations to empower LMIC actors from the outset may help mitigate these imbalances and foster more equitable partnerships.

Despite the clear benefits of these collaborations, several challenges remain. Ethical tensions were also central to participants’ concerns, ranging from disparities in resource access to the financial and emotional burden placed on visiting clinicians. Crowe et al. argue for greater institutional responsibility to buffer the personal toll on individuals engaged in global health partnerships (Crowe et al., 2020). Without structured support, well-intentioned clinicians may suffer burnout or face insurmountable logistical barriers, threatening the continuity of these efforts.

Limited funding, sociocultural differences, and ethical concerns frequently limit the initiation, development and maintenance of such collaborations (Almeida et al., 2018; Timothy et al., 2022). Transferring inputs and models from other specialties to the neurosurgical field may be beneficial to overcome some of such barriers (Crowe et al., 2020).

International neurosurgical collaborations are shaped by institutional and regional differences, which influence how partnerships are formed and sustained. Almeida et al. described models of collaboration between North American and African institutions that emphasized shared surgical training and academic development. However, the structure of healthcare systems, availability of resources, and institutional priorities differ markedly between regions. European partnerships, in particular, may involve more public-sector institutions and stricter administrative processes. This underscores the need for region-specific strategies tailored to the operational context of European institutions (Almeida et al., 2018).

This study contributes to the growing evidence base by offering nuanced insights into the factors driving effective global surgery collaborations that may be extended beyond the field of neurosurgery. First, collaboration initiatives should prioritize building personal trust and long-term connections under the umbrella of a joint vision. Second, collaborations should focus on capacity building aligned with the local context and needs. This should include clinical and academic training tailored to available resources and the healthcare environments. Third, an ongoing emphasis on sustainability should be ensured and efforts directed towards independence. Finally, continuous communication and adaptation to the circumstances are crucial for ensuring the long-term sustainability and impact of the collaborative effort.

While this study does not aim to provide direct prescriptive guidance, the themes and insights herein lay the groundwork for future evidence-based recommendations. The authors are currently engaged in developing practical guidelines based on the data presented here, in collaboration with institutional stakeholders and international partners.

Future research should extend this work by including diverse professional roles within global neurosurgical teams, thereby providing a more comprehensive understanding of collaborative dynamics. Comparative studies exploring HIC partnerships beyond Europe may help evaluate the transferability of our findings. Additionally, prospective longitudinal research could track partnerships from initiation to maturation, identifying key inflection points and sustainability strategies. Finally, developing practical, experience-based guidelines for neurosurgical collaboration—endorsed by major societies—could serve as a valuable resource for institutions embarking on new international initiatives.

## 9 Conclusions

In this study, several key interpersonal and systemic factors that influence the initiation, development, and maintenance of collaborations between European neurosurgical departments and LMIC institutions were highlighted. Trust, local context, capacity building, sustainability, and continuous communication are the pillars on which such collaborations should be based.

These findings are intended to inform future practice-oriented frameworks rather than serve as immediate procedural guidance. Practical recommendations derived from this data are under development and will be addressed in subsequent publications.

## 10 Strengths and limitations

This study presents several strengths. First, it employs a bilateral approach by capturing perspectives from both European neurosurgeons and their LMIC counterparts, which is relatively underrepresented in the existing literature. Second, the study focuses on collaborations of at least five years in duration, allowing insight into long-term dynamics and sustainability. Third, the thematic structure developed through rigorous double-coding and validation enhances the analytical robustness of the results.

However, certain limitations must be acknowledged. While appropriate for a qualitative study, the relatively small and specialized sample size may not fully capture the diversity of global neurosurgical collaborations. Participants were selected based on their sustained involvement in collaborations exceeding five years, potentially introducing a positive bias toward successful experiences. The decision to interview only neurosurgeons was based on their leadership role in initiating and sustaining such collaborations; nonetheless, future studies should include a broader range of professionals (e.g., anesthetists, nurses, administrators, NGO coordinators) for a more holistic view. Additionally, while the study includes partners from different geographical regions, the predominance of collaborations involving European and African institutions may limit generalizability to partnerships involving American, Asian, or Latin American partners.

## Ethics approval and consent to participate

The study was conducted in accordance with the ethical principles of the Declaration of Helsinki. Ethical approval was obtained (GHC250324). Informed consent was obtained from all participants prior to data collection, and participants were assured of confidentiality and their right to withdraw from the study at any time without consequence. All interviews were conducted with participants' explicit consent, and their anonymity was maintained throughout the research process.

## Consent for publication

Not applicable.

## Availability of data and materials

The datasets used and/or analyzed during the current study are not publicly available due to sensitive information; however, they are available from the corresponding author on reasonable request.

## Authors' contributions

Conception and design: NM, VB, EU, MT.

Acquisition of data: NM, VB.

Analysis and interpretation of data: NM, VB, EU, MT; all authors.

Manuscript draft: NM, VB.

Critical revision for important intellectual content: MT, EU; all authors.

Final approval: NM, VB, MT, EU; all authors.

## Funding

This research received no specific grant from any funding agency in the public, commercial, or not-for-profit sectors.

## Declaration of competing interest

The authors declare the following financial interests/personal relationships which may be considered as potential competing interests: The corresponding author is serving, at the time of submission, in the Editorial Board of Brain and Spine as a Reviewer Board member If there are other authors, they declare that they have no known competing financial interests or personal relationships that could have appeared to influence the work reported in this paper.
