# Supplementary material for: Interpersonal and systemic factors in initiating, developing and maintaining collaborations between European neurosurgical departments and institutions in low-resources settings: A qualitative study
Source: Brain Spine. 2025 Jun 19;5:104303. doi: 10.1016/j.bas.2025.104303 (PMC12246693; doi:10.1016/j.bas.2025.104303)
Supplement: Multimedia component 1 [file mmc1.docx]

**Interpersonal and Systemic factors in Initiating, Developing and Maintaining Collaborations between European Neurosurgical Departments and Institutions in low-resources settings: A Qualitative study**

**Research question:** To comprehensively explore interpersonal and systemic factors relevant for the a) initiation, b) development, and c) maintenance of effective and sustainable collaborations between European neurosurgical departments/organizations and LMICs institutions/organizations.

**Semi-Structured Interview Guide**

**Introduction**

*Thank you for agreeing to participate in this research interview. My name is…, I am a Neurosurgeon and I currently work in…*

*The purpose of this interview is to explore the factors that may be relevant to initiating, developing, and maintaining collaborations between European neurosurgical departments/organizations and institutions and organizations in low- and middle-income countries (LMICs). According to your background in this field, you have been purposely identified by the research team as a possible source of precious information and we believe your insights will contribute significantly to understanding the dynamics and challenges of partnerships between European and LMIC institutions.*

*Before we begin, I want to assure you that your responses will be kept confidential and used for research purposes only. The conversation will be recorded and a qualitative analysis will be conducted on its transcripts.*

*During the interview, I ask you to feel as comfortable as possible and to speak freely about your personal experience, including expressing views that are critical of nature. Finally, if there are questions you prefer not to answer, or you at any time want to withdraw from the interview, you are of course free to do so without having to provide any reason for it.*

*The following interview is structured in 3 main sections: after some introductory questions, I will first ask you some questions about the initiation of the collaboration. I then have some questions about how the project developed and finally how it has been maintained. For each section, I will guide you through general and some specific questions.*

*Do I have your consent to process this methodology? Do you have any questions before we proceed?*

*--------------------------------------------------------------------------------------------------------------*

**Background Information:**

*Before I begin with specific questions, I would like to have some general information about yourself and your background:*

1. Can you please introduce yourself and provide a brief overview of your background and current role in neurosurgery?
2. Can you please give a general overview of the collaboration between your institution and the counterpart?

**Initiation Phase:**

*I will now move to some questions that explore the initiation of your collaboration. When responding to the following questions, you should focus at the very initial stages of the partnership.*

1. How did the collaboration between your institution and the counterpart initially come about?
2. Can you describe the key stakeholders involved in the initiation phase? How were they identified and engaged?
3. Were there any formal agreements or frameworks established during the initiation phase to guide the collaboration's direction and objectives? If so, can you please describe them?
4. What were the initial challenges or barriers encountered during the initiation of the collaboration, and how were they addressed?
5. If you could go back, would you do something differently in the initiation of the collaboration? If yes, what would you have done differently?

**Development Phase:**

*I will now move to some questions that explore the development of your collaboration with the counterpart. When responding to the following questions, you should focus at the intermediate stages of the partnerships, when it started to grow and progress*

1. How was the collaboration between your institution and the counterpart structured and organised as it progressed?
2. What strategies were used to develop trust and mutual understanding between the partnering institutions and their respective teams?
3. Can you tell me about any capacity-building initiatives or knowledge transfer activities that were implemented during this phase?
4. What were some of the key milestones or achievements reached during the development phase of the collaboration?
5. You previously mentioned some barriers and obstacles in the initiation of the project. Are the additional barriers encountered while the partnership was developing? How have they been addressed?
6. Were there any instances where the collaboration had to adapt or change its approach? If so, could you provide examples?
7. If you could go back, would you do something differently in the development of the collaboration? If so, could you provide examples?

**Maintenance Phase:**

*I will now move to some questions that explore the maintenance of your collaboration with the counterpart. When responding to the following questions, you should focus at the very late stages of the partnerships*

1. What efforts are made to ensure the sustainability of your collaboration between your institution and the counterpart?
2. Have any changes or adaptations been made to the collaboration over time to meet the evolving needs of both parties better? If yes, how did you/do you assess the evolving needs of both parties? (probe:i.e. feedback from participants or discussion rounds? Other?)
3. You previously mentioned some barriers and obstacles in the initiation and development of the project. Are the additional barriers encountered at this later stage? How have they been addressed?
4. How do you manage potential power imbalances or resource disparities between the partners to ensure equitable participation and decision-making?
5. How does your collaboration cultivate bidirectionality? Can you give some practical examples?

**Reflections and Lessons Learned:**

1. Do you believe that your collaboration helped to develop or improve the autonomy of local collaborators concerning the primary interest of the collaboration? Could you provide some examples?
2. Looking back, How would you describe your collaboration? A joint product? A hierarchical model? Other?
3. Looking back, what are the most significant lessons learned from your experience in initiating, developing, and maintaining collaborations between your institution and the counterpart?
4. According to your experience, what can go wrong in the initiation, development and maintenance of global collaborations? Give some specific experiences
5. Can you share any anecdotes or experiences that particularly stand out to you as illustrating the value or impact of collaboration on patient care or healthcare delivery in the LMIC setting?
6. What advice would you give to individuals or institutions looking to establish similar collaborations? *(probe: please list the 3 key components)*

**Conclusion:**

1. Is there any additional information or insights you would like to share regarding your experiences with collaborative initiatives between European and LMIC neurosurgical departments?

2. Finally, do you have any questions or comments for us before we conclude the interview?

**Closure:**

*Thank you once again for your time and valuable insights. If you have any further questions or wish to follow up on any aspect of this interview, please feel free to contact dr Nicolò Marchesini or dr Vicki Butenshon. If, in a few weeks or so, there are some parts of the interview that I would like to know more about or ask some follow-up questions about, can I arrange a shorter second interview with you?*
